# Civil society participation in global public private partnerships for health

**DOI:** 10.1093/heapol/czy070

**Published:** 2018-08-28

**Authors:** Katerini Tagmatarchi Storeng, Antoine de Bengy Puyvallée

**Affiliations:** 1 Centre for Development and the Environment (SUM), University of Oslo, Postboks 1116, Blindern, Oslo, Norway; 2 Faculty of Epidemiology and Population Health, London School of Hygiene & Tropical Medicine, Keppel Street, London, UK

**Keywords:** Governance, global health policy, nongovernmental organizations, civil society

## Abstract

The growth of global public-private partnerships for health has opened up new spaces for civil society participation in global health governance. Such participation is often justified by the claim that civil society organizations, because of their independence and links to communities, can help address democratic deficits in global-level decision-making processes. This article examines the notion of ‘civil society engagement’ within major public–private partnerships for health, where civil society is often said to play a particularly important role in mediating between public and private spheres. How do major global health partnerships actually define ‘civil society’, who represents civil society within their global-level decision-making bodies, and what formal power do civil society representatives hold relative to other public and private-sector partners? Based on a structured analysis of publicly available documents of 18 of the largest global public–private partnerships for health, we show that many of them make laudatory claims about the value of their ‘civil society engagement’. Most use the term ‘civil society’ to refer to non-governmental organizations and communities affected by particular health issues, and state that they expect these actors to represent the needs and interests of specific populations in global-level decisions about strategies, funding models and policies. Yet, such civil society actors have a relatively low level of representation within the partnerships’ boards and steering committees, especially compared with private-sector actors (10.3 vs 23.7%). Moreover, there is little evidence of civil society representatives’ direct and substantial influence within the partnerships’ global-level governing bodies, where many decisions affecting country-level programmes are made. Rather, their main role within these partnerships seems to be to implement projects and advocate and raise funds, despite common discourses that emphasise civil society's watchdog function and transformative power. The findings suggest the need for in-depth research into the formal and informal power of civil society within global health governance processes.

Key Messages

- Most global public–private partnerships for health emphasize the importance of civil society engagement, but few precisely define civil society or specify civil society’s role within governance processes.
- International NGOs are the main representatives of civil society within health partnership’s global-level governing bodies. They have relatively little formal power compared with private–sector partners.
- Global public–private partnerships emphasize civil society partners’ role in project implementation, advocacy and fundraising, but rarely their role as watchdogs or critical voices in global-level decision-making.

## Introduction

The growth of governance beyond the nation state has been described as ‘one of the most distinct political developments of the past half-century’ (Bexell *et al.*, 2010, p. 81). Perhaps nowhere is this development illustrated more clearly than in the transition from ‘international’ public health to ‘global’ health (Brown *et al.*, 2006). In the new millennium, the post-War system of intergovernmental collaboration overseen by the World Health Organization (WHO) has ceded to a fragmented system of global health governance, defined as ‘the formal and informal institutions, norms and processes which govern or directly influence global health policy and outcomes’ (Sridhar, 2009, p. 1366, Lee and Kamradt-Scott, 2014). Although there is a long history of philanthropic and civil society involvement in health (Birn, 2014), the role of non-state actors including private philanthropy, corporations, and civil society organizations has accelerated and expanded since the late 1990s. Such actors now operate alongside or as part of a dizzying array of public–private partnerships that address specific global health challenges through joint decision-making among multiple partners from the public and for-profit and non-profit private sectors (Buse and Harmer, 2007).

Global public–private partnerships for health have gained traction over the past two decades, enabled by the rising influence of private philanthropic power (notably the Bill & Melinda Gates Foundation) and the incursion of a business-oriented ethos throughout the field of global health (Birn, 2009). Today, these partnerships address global public health challenges ranging from HIV/AIDS to road traffic accidents, malnutrition and lack of access to vaccines and other health commodities. Though widely considered an innovative form of decision-making, critics have been concerned about power imbalances within this new form of governance that are eclipsed by the use of the term ‘partnership’ (Taylor, 2018). In the early years of partnerships like the Global Fund to Fight AIDS, Tuberculosis and Malaria and Gavi, the Vaccine Alliance, Buse and Harmer (2004, p. 49) noted that:

*a northern elite wields power through its domination of governing bodies and also through a discourse which inhibits critical analysis of partnership while imbuing partnership with legitimacy and authority*.

Inviting civil society representatives into public-private partnerships and giving them a seat at the ‘high table’ of global health (Smith *et al.*, 2016) has been a way to respond to such critiques. Political scientists claim that civil society actors can fill the ‘accountability gaps’ in global governance that result from lack of mechanisms like elected leadership and parliamentary oversight, which ensure accountability in nation states (Scholte, 2004, p. 212). As the Lancet’s editor has observed, many within the global health field similarly rely on civil society organizations to provide ‘essential voices in a discordant global health conversation often dominated by risk-adverse multilaterals, corrupt governments and neo-colonial donors’ (Horton, 2016, p. 1041). Other commentators have recently argued that civil society will play a key role in catalysing the transformations needed to attain the United Nations’ 2030 Agenda for Sustainable Development (the SDGs)—by proposing novel policy alternatives, building alliances across sectors and increasing the legitimacy and accountability of global governance processes (Smith *et al.*, 2016). Such optimism is premised on the assumption that civil society organizations’ independence and links to the populations and communities most affected by specific health challenges confers legitimacy and even helps to democratize global governance processes (cf. Scholte, 2004, p. 212).

Although the value of civil society participation is almost ubiquitously endorsed, there is little empirical research into the nature of such engagement within contemporary global health governance mechanisms and processes (Lee, 2010; Kapilashrami and O’Brien, 2012; Smith *et al.*, 2016; Smith, 2017). An emerging body of research from global partnerships ‘country-level’ operations reveals complex power dynamics that limit the extent of true participation (Spicer *et al.*, 2011; Kapilashrami and O’Brien, 2012; Harmer *et al.*, 2013). However, little current literature engages empirically with civil society engagement at the ‘global’ level (see Smith, 2017), where decisions about strategy, funding models and policies affecting partnerships' target countries are primarily made.

To begin to fill this gap, we examine the notion of ‘civil society participation’ within the largest global public–private partnerships for health. Specifically, we analyse how specific partnerships actually define ‘civil society’ and what functions civil society ‘partners’ are expected to fulfil within global-level decision-making processes. Who actually represents civil society within the partnerships' governing bodies and what formal power do these representatives have relative to other partners? Addressing these questions can help us interrogate the meaning of civil society participation within global governance processes and to identify the power dynamics that shape agenda-setting and decision-making.

## Methods

We analysed civil society engagement within 18 global public–private partnerships for health (Table 1), focussing on their operations at the global headquarter level, rather than at the country-level. For convenience and to allow comparison with existing literature, we adopted a list compiled by Hawkes *et al.* (2017) in their recent analysis of gender dynamics within such partnerships. This list—established through a literature review, analysis of official documents and communication with experts—includes those partnerships that operate at the global level, focus on global health issues and have a governance structure engaging both public and private sectors (Buse and Harmer, 2007). Selected partnerships focus on a wide range of topics, such as the development of vaccines, the promotion of handwashing and the use of clean cook stoves, or road safety. These partnerships are headquartered in the main global centres of power, including Geneva and its region (*n =* 11), Washington DC and its region (*n =* 3), New York City, Ottawa, Seoul and Tokyo (*n =* 1) and were founded primarily in the late 1990s to early 2000s (Table 1).

**Table 1.** Description of the 18 global public-private partnerships for health studied

Name	Year of initiation	Focus area	Main decision- making body	Number of seats with voting rights	Headquarters location
Aeras	2005	Vaccines against tuberculosis	Board of directors	11	Rockville, Maryland, USA
DNDi	2003	Drugs for neglected diseases	Board of directors	13	Geneva, Switzerland
Foundation for Innovative New Diagnostics	2003	Diagnostic solutions	Board of directors	12	Geneva, Switzerland
International Vaccine Institute	1997	Develop new vaccines	Board of trustees	14	Seoul, South Korea
Medicines for Malaria Venture	1999	Drugs for malaria	Board of directors	14	Geneva, Switzerland
TB Alliance	2000	Drugs for tuberculosis	Board of directors	7	New York City, NY, USA
Global Alliance for Improved Nutrition (gain)	2002	Malnutrition	Board of directors	13	Geneva, Switzerland
Global Health Innovative Technology Fund	2013	Research and development on infectious diseases	Board of directors	11	Tokyo, Japan
Global Road Safety Partnership	1999	Road safety	Steering committee	15	Geneva, Switzerland
SUN	2010	Malnutrition	Executive committee	15	Geneva, Switzerland
The Global Alliance for Clean Cook Stoves	2010	Air quality	Leadership council	10	Washington, D.C., USA
The Global Handwashing Partnership	2001	Handwashing / WASH	Steering committee	9	Washington, D.C., USA
Nutrition International	1992	Malnutrition / micronutrients	Board of directors	12	Ottawa, Ontario, Canada
Roll Back Malaria	1998	Malaria	Partnership board	14	Vernier, Switzerland
The Global Fund to Fight AIDS, Tuberculosis and Malaria (The Global Fund)	2002	HIV/AIDS; Tuberculosis; Malaria	Board	20	Geneva, Switzerland
The PMNCH	2005	Maternal & child health	Board	29	Geneva, Switzerland
Gavi, The Vaccine Alliance	2000	Vaccines	Board	28	Geneva, Switzerland
Stop TB Partnership	2000	Tuberculosis	Board	25	Vernier, Switzerland

We based our analysis on a structured review of documents that are publicly available on the partnerships’ own websites, conducted in October and November 2017. These documents include web pages describing the partnerships' governance structure, partners, board composition and business model, and pages describing the role of civil society. When available, we also analysed relevant documents linked to from these pages such as the most recent annual reports, strategic plans and board meeting summaries, as well as documents specifically detailing civil society or community engagement. To limit the chances of missing relevant information, we also used the search toolbar on these websites with keywords such as ‘civil society’, ‘NGOs’ (non-governmental organizations), ‘representation’, ‘partners’ and ‘community’. All the documents we selected related to the partnerships’ operations at the global level, though some also mentioned country-level activities.

From these documents, we first extracted explicit and implicit definitions of ‘civil society’ and descriptions of civil society actors’ roles (Table 2). We then analysed the composition of the 18 partnerships’ main decision-making bodies—whether ‘board’, ‘executive committee’, ‘leadership council’ or ‘steering committee’. We first classified board members in the following categories (based on their current or most recent institutional affiliation): (i) ‘NGOs, affected communities, and patient representatives’; (ii) ‘academia and research institutes’ (iii) ‘private sector’; (iv) ‘donor governments’; (v) ‘recipient countries’; (vi) ‘international organizations’; (vii) ‘foundations’; and (viii) ‘other’ (i.e. the partnerships’ executive directors, representatives of other partnerships, a journalist and two trade unions representatives). We then calculated the average representation of these different groups throughout the 18 partnerships. Finally, because most partnerships described ‘NGOs, affected communities, and patient representatives’ as representing ‘civil society’, we examined this category to identify the background and institutional affiliation of the 31 board members within this group.

**Table 2.** Actors included in partnerships’ definitions of ‘civil society’

	Affected communities/ patient representatives	NGOs	Advocacy groups	Academia and research entities	Professional organizations	Faith-based organizations
SUN	X	X	X	X	X	
The Global Fund	X	X	X			X
GAVI	X	X	X	X	X	X
Stop TB Partnership	X	X				

## Findings

### Partnerships' definitions of ‘civil society’

Almost all of the 18 partnerships surveyed refer to the term ‘civil society’ on their websites and in official documents, and all of them describe some level of engagement outside the strictly public or for-profit private sectors. However, only the Global Fund explicitly defines the term ‘civil society,’ specifying that it designates

*all those stakeholders who are neither government bodies nor private sector enterprises: groups such as non-governmental organisations, advocacy groups, faith-based organisations, networks of people living with the diseases, and so on* (Global Fund, 2017a).

NGOs and ‘affected communities’ are also explicitly identified as ‘civil society’ by three of the other partnerships [Gavi, the Vaccine Alliance, Scaling up Nutrition (SUN), and Stop TB partnership] (Table 2), who also variably include ‘advocacy groups’, ‘professional organizations’, ‘faith-based organizations’ and ‘academia and research entities’ within the term. Many other partnerships also similarly mention ‘NGOs and community-based organizations’ (Global Handwashing Partnership, 2017), ‘NGOs and women’s cooperatives’ (Global Alliance for Clean Cook Stoves, 2017a) or ‘NGOs and civil society organizations’ (Gain, 2017) as key ‘partners’, even if they do not explicitly identify them as ‘civil society’. Although some include academia and other research entities within their functional definition of civil society, most partnerships use the term to describe NGOs and ‘affected communities’; the former often representing the latter in practice. The terms NGO, civil society and ‘civil society partners’ are frequently used interchangeably.

### How do public-private partnerships describe civil society roles and functions?

Although their definitions of ‘civil society’ are often vague, many of the partnerships emphasize the value of civil society participation in their work. The Global Fund, for example, claims that, ‘civil society organizations have been at the heart of everything the Global Fund does from the beginning’ (Global Fund, 2017a). Similarly, the board of Stop TB

*recognies that civil society, including affected communities and NGOs, have been critical in catalysing and shaping national, regional and global interventions to scale-up the global TB response* (Stop TB Partnership, 2017a, p. 7).

**Figure 1.**
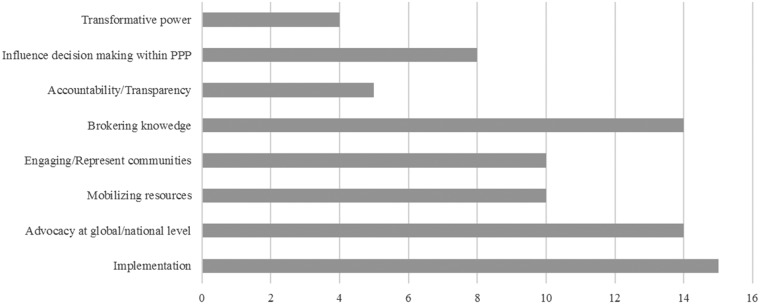
Partnerships' descriptions of civil society partners’ role

Even those partnerships who do not explicitly define ‘civil society’ describe a variety of civil society roles and functions (Figure 1).

The most commonly specified role is as implementing partners at the country level (*n = 15*). Civil society partners are said to play a ‘crucial’ (Gain, 2017), ‘key’ (Gavi, 2017a) or ‘major’ (Find, 2017) role in service delivery, and they receive grants to fulfil this function. At the same time, many also emphasize civil society’s advocacy role (*n = 14*). For example, according to Stop TB’s annual survey of its partners (77% of whom are listed as NGOs)—advocacy is the most common activity, conducted by 84% of partners (Stop TB Partnership, 2017b).

According to the documents reviewed, advocacy can both raise awareness about the partnership’s issue and raise political and donor support. To this end, many partnerships expect their civil society partners to play a critical role in resource mobilization and fundraising on behalf of the organization (*n = 10*). For instance, the Global Fund Advocacy Network, composed mainly of NGOs, aims to create a

*global social movement to demand health for all by recruiting, connecting and mobilising advocates to communicate the urgent need, and demand ‘full funding for the Global Fund’ to maximize its impact* (Global Fund Advocacy Network, 2017, emphasis added).

In addition, several partnerships (the International Vaccine Institute, Drugs for Neglected Diseases Initiative [DNDi], SUN, the Global Alliance for Clean Cook Stoves, the Global Fund and Stop TB) have set-up ‘support committees’, ‘champions’, ‘ambassadors’ or ‘friends’ networks to mobilize high-level individuals, including some from ‘civil society’ such as artists and celebrities, to advocate and raise funds for the partnerships. For example, the Global Fund, in addition to its advocacy network, has established five active regional independent associations known as ‘Friends’, who ‘help develop contacts and allies, promote a good understanding of the Global Fund mission, and mobilize political and financial support’ (Global Fund, 2017b). One of the specified selection criteria for board membership of the Friends groups is to have ‘capacity to mobilize resources.’ The board includes many prominent individuals, like famous musicians from sub-Saharan Africa (e.g. Angelique Kidjo and Youssou N’Dour), development experts from high-income countries (e.g. Jeffrey Sachs), and for-profit private sector leaders (e.g. from De Beers Consolidated Mines).

Moreover, most of the partnerships expect their civil society partners to broker knowledge (*n = 14*), which often implies providing important feedback from the ground to ensure effective implementation of partnership-funded programmes. The partnership structure is said to afford civil society actors the possibility to ‘exchange best practices’ (Global Alliance for Clean Cook Stoves, 2017b) and ‘share information and expertise for development and scaling up of best practices’ (Roll Back Malaria, 2017). Civil society partners are also said to represent affected communities or patients (*n = 10*), thereby ensuring ‘sustainable, locally owned and managed solutions’ (Global Road Safety Partnership, 2017), helping to ‘reach the unreached’ whilst being ‘responsive to the need of communities’ (Gavi, 2017a). In brief, they are expected to ensure that the ‘needs and interests of key populations’ are represented (Global Fund, 2017a).

Despite this emphasis on representation and ‘expert local knowledge’ (Gain, 2017), less than half of the partnerships explicitly state that civil society has a role in influencing decision-making at the global level (*n = 8*). Even then, they often talk not exclusively about civil society representatives but more generally about ‘partners’ and make ambiguous statements like ‘civil society has played an active role in policy development’ (Global Fund, 2017a), or ‘partners contribute through participation in strategy and policy-setting’ (Gavi, 2017b). Finally, only some describe civil society as a transformative power (*n = 4*), in, for instance, ‘question[ing] the status quo and demand[ing] change’ (Global Fund, 2017a) and ‘bringing the voice of change and societal improvement’ (Global Road Safety Partnership, 2017). Similarly, only some (*n = 5*) explicitly refer to civil society’s ‘watchdog’ function (Global Fund, 2017a; Stop TB Partnership, 2017c) in ensuring accountability and transparency, whether in governance processes or in programme implementation.

### Civil society representation in partnerships’ governing bodies

**Figure 2.**
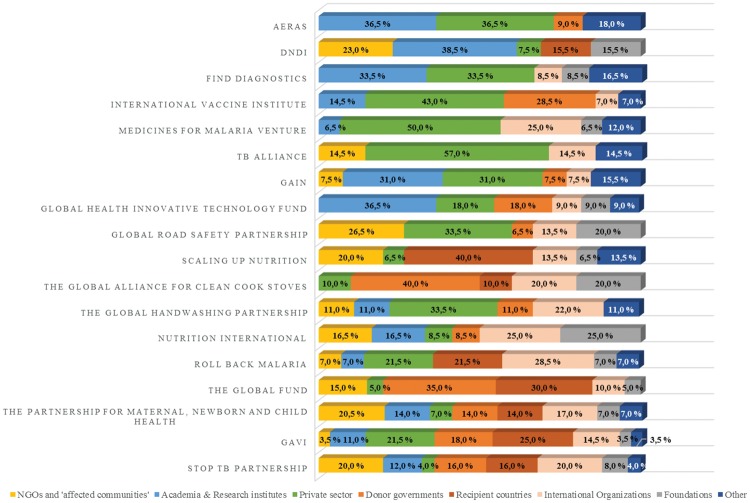
Partnerships' board composition (November 2017)

The impression that civil society partners’ role is largely to support the partnerships rather than to shape or challenge decision-making is reinforced by their relatively low level of representation within formal decision-making bodies (Figure 2). Several of the largest partnerships (SUN, the Global Fund, Stop TB, the Partnership for Maternal, Newborn and Child Health [PMNCH], Gavi and Roll Back Malaria) have civil society board members who are elected from within dedicated civil society ‘constituencies’, which can include between a dozen and hundreds of organizations (e.g., 472 for the PMNCH’s NGO constituency) (PMNCH, 2016). They often have dedicated personnel to ensure coordination and continued activities (a ‘constituency focal point’). The Global Fund has perhaps the most sophisticated arrangements for civil society engagement; with one Board member seat each for Developed Country NGOs, Developing Country NGOs and Communities Living with the Diseases Delegations. However, most partnerships' civil society board members are recruited in their individual capacity, with no or imprecise selection criteria. As the SUN Movement states: ‘membership will reflect, but not represent, the diverse countries, organizations and networks of the Movement’. Although board members are not meant to ‘represent’ viewpoints in a strict sense, they are however clearly selected to bring different perspectives to the table.

Because the partnerships most often use the term ‘civil society’ to describe NGOs, affected communities and patient representatives, we measured these groups’ level of representation in the governing bodies. Among the 274 individuals represented on the boards of the 18 partnerships we surveyed, only 31 represent affected communities or NGOs. The level of representation varies greatly, ranging from no representation in four partnerships (Aeras, the International Vaccine Institute, the Medicines for Malaria Venture and the Global Health Technology Fund) to 26.5% of board members in the Global Road Safety Partnership, with an average of 10.3% throughout the sample. The level of civil society representation increases significantly if the definition incorporates academia (25.2%), though this encompasses actors whose primary functions are described as aiding product development rather than representing the interest of people or patients.

**Figure 3.**
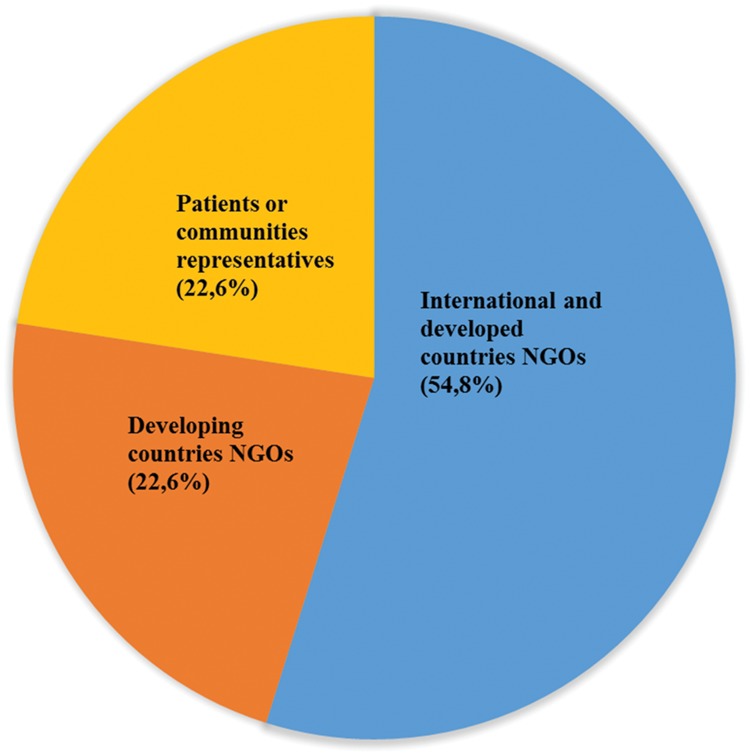
Institutional affiliation of the partnerships' board members representing ‘NGO and affected communities’

Three-quarters of the individuals representing NGOs and affected communities are from international NGOs working with partnerships to implement grants and programmes; less than a quarter (22.6%) are ‘patient’ or ‘affected communities’ representatives (Figure 3). In real terms, this means that only 7 out 274 seats (2.6%) across the 18 partnerships are held by individuals speaking on behalf of the millions of patients and others directly affected by the diseases and health issues they target. Two of these seven seats are held by medical doctors classified as ‘patients advocates’ (DNDi) rather than as representatives of civil society organizations for affected individuals, while the PMNCH’s ‘adolescents and youths’ representative is a 22-year-old Botswanan woman with a large philanthropic endowment (Botswana Youth, 2013).

**Figure 4.**
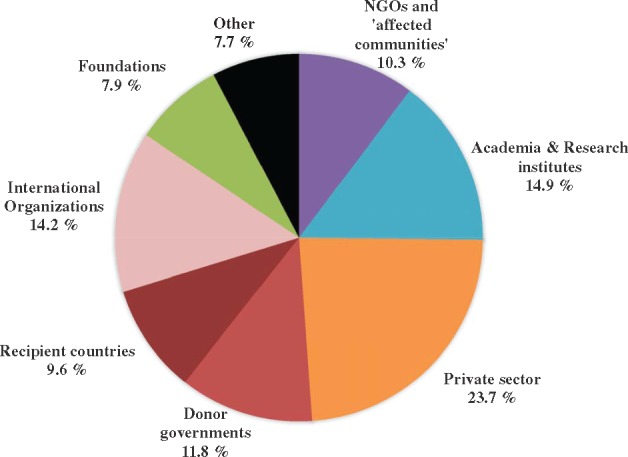
Average board composition of 18 major public–private partnerships for global health

NGOs and affected communities’ combined representation within the decision-making bodies (10.3% of the voting seats) puts them in a weak position relative to other partners. Academia and research institutes (14.9%), international organizations (14.2%) and donor governments (11.8%) all have higher levels of representation than the group of actors meant to speak on behalf of the intended beneficiaries of the partnerships’ programmes (Figure 4).

Although NGOs and affected communities combined have slightly higher levels of representation than recipient countries (9.6%), foundations (7.9%) and ‘others’ (7.7%), they have a far lower level of representation than the private sector, which holds 23.7% of the voting seats across partnerships. Industry representatives and other individuals working in the for-profit private sector dominate many of the boards. They even have the majority of the seats in the Medicine for Malaria Venture (50%) and the TB Alliance (57%) boards. More generally, the private sector tends to have stronger formal influence in partnerships focussed on product development rather than in those having broader system goals, such as the PMNCH (6.9%) or the Global Fund (5%). A similar pattern exists for academia and non-profit research institutes, which hold around a third of the seats in the boards of Aeras, DNDi, Find and the Global Health Innovative Technology Fund—all organizations that finance research to develop biotechnical products.

Analysing the level of representation by constituency masks the influence of specific organizations and individuals across many of the partnerships. Notably, the Bill and Melinda Gates Foundation holds a seat in half (*n = 9*) of the decision-making bodies. This makes it the most widely represented organization, before the WHO (44%, *n = 8*) and the World Bank (33%, *n = 6*). We also found that 15 board members (6.7% of all the individual decision-making body member names collected) participate in at least two governing bodies.

## Discussion

The global public-private partnerships for health that have proliferated since the late 1990s have opened up new spaces for civil society engagement (Kapilashrami and O’Brien, 2012; People's Health Movement and Medact, 2014). Our anaysis of 18 such partnerships shows that most of them indeed describe some level of engagement with actors they label civil society ‘partners’ and emphasise its value. However, our findings confirm the existence of a ‘rhetoric-reality gap’ when it comes to civil society participation (Kapilashrami and Hanefeld, 2014, Storeng and Béhague, 2016).

The term civil society is widely brandished, but rarely explicitly defined. Most partnerships use the term functionally to describe specific actors, primarily NGOs and ‘affected communities’—in keeping with the way in which the term has been described within the global health literature. For instance, Doyle and Patel’s (2008, p. 1928) influential literature review of civil society participation in global initiatives for HIV/AIDS defines civil society organizations to include ‘non-profit making or ‘public-interest’ organizations established by citizens to improve health’. They specify that these are typically NGOs or community-based organizations that ‘either deliver health interventions or lobby for change in policy to tackle global health problems (advocacy NGOs)’ (*ibid.*). A more recent review used the term civil society to refer to ‘voluntary, non-state, not-for-profit organizations formed by people in the social sphere with commonly held values, beliefs and/or causes’ (Smith *et al.*, 2016, p. 1). These definitions largely conflate civil society with NGOs, and are narrower than some of the definitions adopted by international relations scholars and political scientists to conceptualize the notion of ‘civil society’ within contemporary world politics (Hendriks, 2006). Scholte (2011, p. 214), for instance, has defined civil society as ‘a political space where voluntary organizations form outside political parties to shape the rules that govern one or other aspect of social life’. In this reading, NGOs are just one of many different types of civil society organizations—anti-globalization movements, environmental movements, women’s associations and trade unions—trying to influence global regulatory institutions and other aspects of social life (Erman and Uhlin, 2010; Steffek and Hahn, 2010).

Our analysis identified a number of mechanisms with which public–private partnerships for health ‘engage’ civil society at the global level, including by offering formal representation in decision-making bodies and by supporting dedicated advocacy networks and individual advocacy champions who conduct advocacy and fundraising. The Global Fund, was the first partnership to include civil society organizations as decision-makers on its board (Smith, 2017). It also appears to have the most sophisticated architecture for civil society engagement at the global level—with three distinct delegations providing board representatives, a global advocacy network, five associations of ‘Friends’ spread all over the world and an independent NGO, Aidspan, providing external oversight. Most of the partnerships we reviewed also offer civil society representation on their decision-making bodies, though the level of representation varies greatly. Gavi (2017a), for instance, stresses the crucial role NGOs play in the implementation of programmes and in ‘reaching the unreached’, but only grants one out of its board’s 28 seats (3.5%) to an NGO representative, based in Cameroon.

Although some civil society representatives are selected from within specific interest-based constituencies that have their own appointment processes, such as in the Global Fund NGO Delegations, others are prominent personalities chosen for their individual qualities. As Doyle and Patel (2008) have noted, methods for selecting civil society representatives that lack transparency can be problematic, because politically progressive actors may be favoured over more subservient service-delivery NGOs who, because of their financial dependency on donors within the partnership, may be less likely to challenge the status quo.

Indeed, our review suggests that civil society board representatives tend to be ‘partners’ whose main function is to implement programmes and do advocacy on behalf of the partnerships, in keeping with the observation that civil society organizations that represent hegemonic interests rather than presenting alternatives, have greatest influence within global governance forums (Bebbington *et al.*, 2008). Our data, however, do not enable us to conclude whether this bias results from active exclusion or instead from more politically progressive civil society groups’ decision to work outside of formal structures to maintain independence. Many civil society representatives are based in international NGOs, sometimes with weak or indirect links to people affected by the health issues they champion. Many civil society ‘partners’ can be characterized as ‘conformist’ top-down global NGOs rather than more ‘transformative’ bottom-up transnational movements of solidarity and action (cf. Scholte, 2005). Substantial funding from Western donor state, corporate and philanthropic donors has, for example, enabled NGOs like Save the Children and PATH, to establish themselves as ‘professional altruists’ (Mosse, 2011). With headquarters in global centres of power like Geneva, London, New York or Seattle, many international NGOs oversee multi-country programmes that are issue-based rather than representative of specific communities (People's Health Movement and Medact, 2014). More in-depth research is needed to understand the extent to which such civil society partners have the input legitimacy—inclusive deliberation, fair process and transparency—that may be required to address legitimacy and knowledge deficits within global public–private partnerships (Shiffman, 2015).

Despite the frequent emphasis on civil society as core partners, it is striking that NGOs and affected communities—the categories most frequently referred to as ‘civil society’—have relatively low levels of representation relative to other categories of partners, notably private-sector partners. Certain actors, notably the Gates Foundation and prominent policy specialists, recur across a number of the partnerships, suggesting the presence of a strong global epistemic community or power elite. Although civil society has gained formal power through representation on global decision-making bodies, our findings do not substantially challenge Buse and Harmer’s (2014) earlier observation that Northern policy-making elites dominate global public–private partnerships for health. Indeed, recent ethnographic studies identify philanthropic partners and technical experts operating at a global level as the most powerful actors within such configurations (Storeng, 2014; Storeng and Béhague, 2016).

Although our analysis suggests that civil society partners’ formal power is low relative to other partners, measures of representation do not provide evidence of the level of ‘actual’ influence; what civil society engagement means in practice is difficult to discern from the documents we have reviewed. Many of today’s leading partnerships make laudatory claims about the value of ‘engaging’ civil society. Gavi (2017a), for instance, credits civil society with reaching the unreached, contributing to health system strengthening, influencing public policy, supporting resource mobilization and enhancing transparency and accountability. Yet, the partnerships’ publicly available documents do not provide evidence of direct and substantial impact on the partnership’s global decision-making. Rather, civil society partners' main role seems to be to advocate and raise funds for the partnerships, as well as discuss best practices to implement programmes more effectively. We cannot, then, take at face value claims by leading partnerships like Gavi, that by participating in its governance at the global level, civil society organizations ‘provide input to ensure that its programmes and policies are robust and that the Vaccine Alliance maintains a high level of transparency, accountability and responsiveness’ (Gavi, 2017a).

Interrogating such claims requires further in-depth analysis using more open-ended methods. This can include ethnographies of expert communities, observations of civil society representatives’ interactions with other board members, critical review of board meeting minutes and in-depth interviews. Such research must pay attention not just to the ‘formal’ power afforded to civil society partners through representation on boards, which we analyse here, but also more informal mechanisms of influence (Smith, 2017). As Florini (2008, p. 687) argues, ‘the power of INGOs (international NGOs) and other manifestations of transnational civil society is a soft, diffuse power, one that shapes norms and ideas in crucial ways that is often not reflected in formal power structures’.

Informal modes of influence resulting from normative or epistemic power (Hanefeld and Walt; 2015, Shiffman, 2015) may occur not just at the global level, which we have analysed, but also at the interface between global and national-level policy processes and politics, especially because many of the international NGOs who work to set norms, policies and aspirations at the global level, simultaneously support struggles for health at national and local levels (Birn, 2008). As global partnerships exert ever more influence over the policy space in many low- and middle-income countries, understanding the linkages between their civil society engagements at the global and national levels thus becomes imperative. Several of the partnerships we have reviewed make significant efforts to create and strengthen networks of civil society organizations at the national and local levels, often with the dual objective of advocacy and resource mobilization and co-ordinating programme implementation. For example, the Global Fund aims to ‘create ways for civil society to more deeply understand the current state of domestic financing for health, including as those trends relate to countries’ co-financing commitments to the Global Fund’ (Global Fund, 2017a). These networks also aim to ‘oversee the implementation of approved grants’ and ‘ensure linkages and consistency between Global Fund grants and other national health and development programmes’ (Global Fund 2017c).

However, recent, in-depth analyses suggest a range of constraints on meaningful participation in such country-level initiatives. In India, the notion of civil society within in the Global Fund’s country-coordinating mechanisms actually ‘underplays differences and power dynamics between various institutions and conceals the agency of outsiders under the guise of autonomy of the state and people’ (Kapilashrami and O’Brien, 2012, p. 437). Similarly, Global Fund support to civil society advocacy efforts to reform HIV/AIDS and drugs-related policies in three former Soviet Union countries had ambiguous effects: professionalization of civil society organizations increased confidence from government, but Global Fund support created competition for funds between different NGOs, causing resentment and weakening capacity for effective, collective advocacy (Harmer *et al.*, 2013).

Empirical research is needed to engage with broader normative debates about whether civil society organizations act as agents of resistance, democratic change and social justice or rather depoliticizes governance at the global level (Watkins *et al.*, 2012; Hannah, 2016). Scholte (2011, p. 313), for instance, argues that civil society interventions have often reinforced arbitrary power hierarchies in global politics and legitimated rather than challenged existing but flawed global governance arrangements. Such arguments are echoed in the empirical literature on civil society engagement in global health governance, where authors have warned that one of the main effects of civil society participation may be to confer an undue legitimacy on the ‘public–private partnership’ model itself (Doyle and Patel, 2008).

## Conclusion

If we understand global health as a complex system of power relations in which actors deploy financial capital, expertise and moral authority to pursue career and organizational interests (Shiffman, 2015), it is clearly important to delineate the social and political dynamics of civil society engagement within this system. This article provides a window onto such dynamics at the global level. What is needed now is better in-depth understanding of whether and how civil society actors participate in developing public-private partnerships’ organizational strategy, designing the funding model, overseeing the work of the secretariat and establishing - and indeed challenging - policy. This requires going beyond the analysis of the formal power they are afforded through representation, as we have done here, and examine the more subtle processes through which civil society actors mobilize more diffuse normative and epistemic power. This implies a need for research into how and why the actors representing ‘civil society’—and those seeking to influence them from outside—come to differ in their values, in how they convene and deliberate, in their financial interests, and indeed in their relationships with donors and their constituents (cf. Medicus Mundi International, 2016). Insights about ‘civil society's’ ability to catalyse social and political transformations is urgent, especially given the widespread assumption that civil society organizations will act as watchdogs to protect global public health within a context in which private-sector actors and interests wield ever more power.
